# The impact of *in situ* breast cancer and family history on risk of subsequent breast cancer events and mortality - a population-based study from Sweden

**DOI:** 10.1186/s13058-016-0764-7

**Published:** 2016-10-18

**Authors:** Helena Sackey, Miao Hui, Kamila Czene, Helena Verkooijen, Gustaf Edgren, Jan Frisell, Mikael Hartman

**Affiliations:** 1 Department of Molecular Medicine and Surgery, Karolinska Institutet, Stockholm, Sweden; 2 Saw Swee Hock School of Public Health, National University of Singapore, Singapore, Singapore; 3 Department of Medical Epidemiology and Biostatistics, Karolinska Institutet, Stockholm, Sweden; 4 Imaging Division, University Medical Center Utrecht, Utrecht, The Netherlands; 5 Department of Surgery, National University Hospital, Singapore, Singapore; 6 Department of Breast and Endocrine Surgery, Karolinska University Hospital, Stockholm, Sweden; 7 Hematology Center, Karolinska University Hospital, Stockholm, Sweden

**Keywords:** *In situ* breast cancer, Mortality, Second event, Contralateral breast cancer

## Abstract

**Background:**

The clinical behavior of *in situ* breast cancer is incompletely understood and several factors have been associated with invasive recurrence. The purpose of this study was to evaluate long-term risk of subsequent breast cancer and mortality among women diagnosed with *in situ* breast cancer, in relation to family history

**Methods:**

Using the population-based Swedish Multi-Generation and Cancer Registers we identified 8111 women diagnosed with *in situ* breast cancer between 1980 and 2004. We used standardized incidence ratios (SIRs) to measure the relative risk of subsequent invasive or contralateral *in situ* breast cancer and standardized mortality ratios (SMRs) for relative risks of death.

**Results:**

Among women diagnosed with *in situ* breast cancer, the cumulative 10-year and 20-year risk for subsequent contralateral or ipsilateral invasive cancer was approximately 10 % and 18 %, respectively. The risk of subsequent invasive breast cancer was increased more than 4-fold (SIR 4.6 (95 % CI 4.2 − 4.9)) among women with *in situ* breast cancer as compared to women in the general population and the risk of contralateral *in situ* breast cancer was increased almost 16-fold (SIR 16.0 (95 % CI 13.2–19.1)). Having a family history of breast cancer increased the risk of contralateral invasive breast cancer by almost 50 % (incidence rate ratio 1.5 (95 % CI 1.0–2.0)). Women under forty years old at diagnosis, without family history, had a 7-fold increased risk, and those with a family history had a 14-fold increased risk for subsequent invasive breast cancer with SIRs of 7.2 (95 % CI 4.8–10.5) and 14.3 (95 % CI 7.4–25.0), respectively. The overall risk of death in women with *in situ* breast cancer was significantly increased by 30 % compared to the general population but was highly dependent on the occurrence of a second invasive cancer event (SMR 1.3 (95 % CI 1.2–1.4)).

**Conclusions:**

Among women with *in situ* breast cancer, a positive family history increases the risk of contralateral invasive breast cancer by almost 50 %. The risk of subsequent invasive breast cancer and mortality is substantially higher in younger women, which should be taken into account when planning their treatment and follow up.

**Electronic supplementary material:**

The online version of this article (doi:10.1186/s13058-016-0764-7) contains supplementary material, which is available to authorized users.

## Background

Women with *in situ* breast cancer have an increased risk of developing *in situ* or invasive breast cancer in the ipsilateral or contralateral breast [1–12]. Moreover women with *in situ* breast cancer, even after treatment, are at increased risk of subsequent invasive breast cancer compared to women in the general population [1, 3–9, 13–16]. The clinical behavior of *in situ* breast cancer is incompletely understood but it is likely that it represents a mixed population of indolent and more aggressive tumors. Several factors have been associated with invasive recurrences, including patient characteristics [4, 5, 8], tumor characteristics [4, 5, 17] and treatment [4, 18, 19]. The influence of a positive family history on subsequent breast cancer is less well-studied [20–22].

The risk of death from breast cancer in women diagnosed with *in situ* breast cancer is considered to be at most only marginally increased, but remains less well-characterized and, with few exceptions, studies are often limited by short follow up and non-population-based designs [14, 23]. In this study we evaluated the long-term risk of second breast cancer and death among women diagnosed with *in situ* breast cancer, in relation to family history.

## Methods

We combined data from the Multi-Generation Register (including more than 11 million individuals, from around 3 million families) with the Swedish Cancer Register, the Cause of Death Register and the Total Population Register for data on emigration. These registers were merged using the unique national registration number that all Swedish citizens receive at birth or immigration. Linkages provide complete follow up with of cancers, vital status, date and cause of death, and dates of immigration and emigration. It also provides links between children and parents through their respective national registration numbers.

The Swedish Cancer Register is a nationwide, population-based register that contains information on virtually all diagnosed cancers in Sweden since 1958 and is considered almost complete for invasive cancer [24–26] and of very high reliability for *in situ* breast cancer from 1980 onwards [26]. The tumor site is classified according to international classification of disease (ICD). Any invasive cancer following *in situ* breast cancer is reported as a new event, as are new *in situ* breast cancers in the contralateral breast. Local relapses are not recorded, neither are new ipsilateral *in situ* events. The register does not distinguish ductal from lobular *in situ* breast cancer before 1990 and contains no information on tumor stage or treatment. Ipsilateral *in situ* breast cancer was excluded due to the increased probability of being underreported in women with previous *in situ* breast cancer.

Thus, we defined subsequent breast events as ipsilateral or contralateral invasive or a contralateral *in situ* breast cancer. Women with any previous invasive or *in situ* breast cancer were excluded, as were women with invasive breast cancer diagnosed concurrently with the first *in situ* breast cancer. Family history of breast cancer was defined as having at least one first-degree relative diagnosed with invasive breast cancer at any point in time. For all women, we collected information on family history of breast cancer and all second primary cancers including type of cancer, laterality and date of diagnosis. Because of incomplete information on laterality and *in situ* breast cancer registration prior to 1980 [26], we restricted our cohort to women with a first *in situ* breast cancer diagnosed in the period 1980 to 2004. Our final study population consisted of 8111 women in the Swedish Multi-Generation Register, diagnosed with first *in situ* breast cancer between 1 January 1980 and 1 January 2005.

### Statistical analyses

#### Risk of subsequent breast events following in situ breast cancer

To estimate the risk of a subsequent breast event (ipsilateral or contralateral invasive or contralateral *in situ* breast cancer), all women were followed from the date of their first *in situ* breast cancer diagnosis and continued until a subsequent breast cancer, emigration, death, or end of follow up, whichever came first. We estimated standardized incidence ratios (SIRs), i.e., the ratio of the observed to the expected number of breast cancers (ipsilateral or contralateral invasive or contralateral *in situ* breast cancer), as a measure of relative risk. The expected number of subsequent breast cancer events was calculated as the product of the person-years accumulated by women with *in situ* breast cancer by the age-specific and calendar-period-specific incidence of unilateral *in situ*/invasive breast cancer in the general population in the Swedish Multi-Generation Register.

For all estimates for the contralateral breast, the background rate of *in situ* and invasive breast cancer was divided by two, as only one breast was “at risk”. Thus, SIRs compare sex-adjusted, age-adjusted and calendar-period-adjusted risk of subsequent events (ipsilateral or contralateral invasive or contralateral *in situ* breast cancer) among patients with *in situ* breast cancer to the risk among the general population, and were stratified by family history of breast cancer. SIRs of subsequent invasive breast cancer were calculated for the calendar period of the first diagnosis, age and time since first diagnosis. Poisson trend tests for monotonic trend of SIRs across calendar period, age and time since first diagnosis was performed [27]. We used Poisson regression modeling among women with a first *in situ* breast cancer to estimate the independent effects of age, year and time since diagnosis and effect of family history on the risk of ipsilateral or contralateral invasive or contralateral *in situ* breast cancer.

As background rates of breast cancer vary considerably by age we also estimated excess additive risks (EARs), as the difference of observed numbers of subsequent invasive breast cancers and the expected numbers in the general population in the Swedish Multi-Generation register, as a measure of absolute risk for subsequent invasive cancer. EARs were estimated using a univariate Poisson model with an identity link function and the expected number of cases as the offset. The likelihood ratio test was used to calculate 95 % confidence intervals (CIs). The cumulative incidence was estimated using the life-table (actuarial) method.

#### Risk of death following in situ breast cancer

The standardized mortality ratio (SMR), i.e., the ratio of the observed to the expected number of deaths, standardized by age and calendar period, was used as a measure of relative mortality. The expected number of deaths was calculated from the general population in the Swedish Multi-Generation register. SMRs were also stratified by family history, age at first *in situ* breast cancer diagnosis and type of subsequent breast event. For overall SMRs, subjects were followed from the date of the first *in situ* breast cancer diagnosis until the date of emigration, death, or end of follow up, whichever came first. In contrast, in the estimates of death by type of subsequent breast event, follow up was started at the diagnosis of that particular event. We calculated 95 % CIs assuming a Poisson distribution for the observed number of cases. All data preparation and analysis was done using the SAS statistical package, version 8.2 or higher (SAS Institute Inc., Cary, NC, USA). The regional ethical committee in Stockholm approved the study.

## Results

Patient characteristics are listed in Table 1. Over a follow-up period of 71,458 person-years, 825 (10.2 %) women developed 886 subsequent breast events (118 contralateral *in situ* and 768 ipsilateral or contralateral invasive breast cancers). The proportion of subsequent breast events was similar in women with and without a family history (11.3 %, n = 97 versus 10.0 %, n = 728). The average time from the first *in situ* breast cancer diagnosis to a second breast event was overall 5.6 years +/− 4.6 years.

**Table 1:** Summary of all women diagnosed with *in situ* breast cancer from 1980 to 2004

	All	No family history	Family history
Total	8111	7252	859
Mean age at first *in situ* breast cancer (SD)	59.1 (12.1)	59.7 (12.1)	53.9 (10.8)
Mean follow-up time, years (SD)	8.8 (5.9)	8.3 (5.9)	7.7 (5.4)
Year at diagnosis of first *in situ* cancer			
1980 − 1984	665	624	41
1985 − 1989	1211	1108	103
1990 − 1994	2046	1835	211
1995 − 1999	1963	1727	236
2000 − 2004	2226	1958	268
Age at diagnosis of first *in situ* cancer, years			
< 40	335	269	66
40–44	594	507	87
45–49	1078	903	175
50–54	1313	1133	180
55–59	1133	993	140
60–64	1021	943	78
65–69	1058	995	63
70–74	778	748	30
75+	801	761	40
Type of second events			
Contralateral *in situ*	118	104	14
Ipsilateral invasive	376	334	42
Contralateral invasive	303	262	41
Total invasive^a^	768	677	91
Second breast event total^a,b^	886	781	105
Type of second events (women, *n*)			
Contralateral *in situ* ^a^	117	103	14
Ipsilateral invasive	370	328	42
Contralateral invasive	299	258	41
Total invasive^a^	725	637	88
Second breast event total^a,b^	825	728	97

^a^Includes the events where laterality is missing. ^2b^ipsilateral *in situ* events were not included in the study

### Risk

Table 2 presents the risk of second invasive or *in situ* breast cancer. The risk of a subsequent ipsilateral or contralateral invasive breast cancer was increased more than fourfold (SIR 4.6 (95 % CI 4.2–4.9)) among women with *in situ* breast cancer as compared to women in the general population. The risk of contralateral *in situ* breast cancer was almost 16-fold increased (SIR 16.0 (95 % CI, 13.2–19.1)). Poisson regression analyses showed that women with a family history of breast cancer had almost 50 % increased risk of contralateral invasive breast cancer, compared to women without a family history of breast cancer (adjusted IRR 1.5 (95 % CI 1.0–2.0)).

**Table 2:** Standardized incidence ratio (SIR) of a second breast event (contralateral *in situ* or ipsilateral or contralateral invasive breast cancer) after diagnosis of first *in situ* breast cancer and its 95 % CI, by type of second breast event and family history

	All		No family history		Family history		Incidence rate ratio^d^
	Number	SIR	Number	SIR	Number	SIR	
		(95 % CI)		(95 % CI)		(95 % CI)	(95 % CI)
Second breast cancer^a^	886	5.1 (4.8–5.4)	781	5.0 (4.6 − 5.3)	105	6.3 (5.1–7.6)	1.2 (1.0–1.4)
Second contralateral *in situ* ^a^	118	16.0 (13.2–19.1)	104	15.8 (12.9–19.2)	14	17.4 (9.5– 29.3)	1.1 (0.6–1.9)
Second invasive^b^	768	4.6 (4.2–4.9)	677	4.4 (4.1–4.7)	91	5.6 (4.5–6.9)	1.2 (1.0–1.5)
Second ipsilateral invasive^c^	376	4.3 (3.8–4.7)	334	4.2 (3.8–4.7)	42	5.0 (3.6–6.7)	1.00 (0.7– 1.4)
Second contralateral invasive^c^	303	3.4 (3.1–3.8)	262	3.3 (2.9–3.7)	41	4.8 (3.5–6.5)	1.5 (1.0–2.0)

^a^Background rate of *in situ* breast cancer was divided by 2. ^bI^includes ipsilateral, contralateral and missing side. ^c^Background rate of invasive breast cancer was divided by 2. ^d^Reference group is *No family history*. Incidence rate ratio has been adjusted for age and year of first diagnosis of *in situ* cancer and time since first diagnosis

**Fig. 1:**
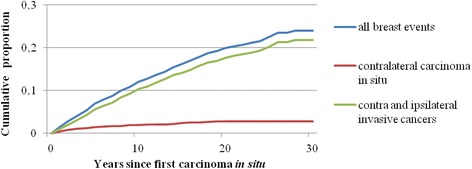
Cumulative incidence of a second breast event among women diagnosed with *in situ* breast cancer, stratified by types of subsequent breast events

Among women diagnosed with *in situ* breast cancer, the cumulative 10-year and 20-year risk of subsequent contralateral or ipsilateral invasive cancer was approximately 10 % and 18 %, respectively, while the cumulative 10-year and 20-year risk of subsequent contralateral *in situ* breast cancer was 1 % and 2 %, respectively (Fig. 1).

Women with *in situ* breast cancer and no family history had increasing risk of subsequent invasive cancer during the study period, with a SIR of 3.1 (95 % CI 2.4–3.9) in 1980–1984, vs. 5.0 (95 % CI 3.9–6.5) in 2000–2004 (*P* for trend <0.001). In contrast, women with a family history did not have increased risk of subsequent invasive breast cancer over the study period (Table 3). The EAR also increased over the study period for women with no family history of breast cancer but did not increase in women with a family history (Additional file 1: Table S1).

**Table 3:** Standardized incidence ratio (SIR) of second invasive breast cancer (ipsilateral and contra lateral) after diagnosis of first *in situ* breast cancer, by year at first diagnosis, age at first diagnosis, time since first diagnosis and family history (years)

	All			No family history			Family history		
	Number	SIR	95 % CI	Number	SIR	95 % CI	Number	SIR	95 % CI
Calendar year^a^									
1980–1984	81	3.3	2.6–4.1	72	3.1	2.4 − 3.9	9	6.6	3.0–12.5
1985–1989	141	3.5	3.0 − 4.2	123	3.4	2.8–4.0	18	5.3	3.2–8.4
1990–1994	292	5.2	4.6 − 5.9	259	5.1	4.5–5.8	33	5.9	4.1–8.3
1995–1999	182	5.2	4.5 − 6.1	160	5.2	4.4–6.0	22	5.2	3.3–7.9
2000–2004	72	5.1	4.0 − 6.4	63	5.1	3.9–6.5	9	5.5	2.5–10.4
*P* value	<0.001				<0.001			1	
Age at diagnosis (years)									
< 40	39	8.5	6.0–11.7	27	7.2	4.8–10.5	12	14.3	7.4–25.0
40–49	173	4.9	4.2–5.7	147	4.8	4.1–5.71	26	4.7	3.1–6.8
50–59	221	4.1	3.6–4.6	189	3.9	3.4–4.5	32	5.2	3.6–7.4
60–69	220	4.6	4.0–5.2	207	4.5	(4.0–5.2)	13	5.2	2.8–8.8
≥ 70	115	4.3	3.6–5.2	107	4.2	(3.4–5.1)	8	6.9	3.0–13.6
*P* trend	0.008				0.069			0.096	
< 40	39	8.5	6.1–11.7	27	7.2	4.8–10.5	12	14.3	7.4–25.0
> 40	729	4.4	4.1–4.8	650	4.3	4.0–4.7	79	5.2	4.1–6.4
*P* value	<0.001				0.012			0.001	
Time since diagnosis (years)									
0–4	401	5.2	4.7–5.7	359	5.1	4.6–5.7	42	5.5	3.9–7.3
5–9	230	4.4	3.9–5.1	197	4.2	3.6–4.8	33	6.5	4.5–9.2
10–14	96	3.4	2.8–4.2	85	3.3	2.6–4.1	11	4.3	2.1–7.7
> 15	41	3.4	2.4–4.6	36	3.2	2.2–4.4	5	5.9	1.9–13.8
*P* trend	<0.001				<0.001			0.848	

^a^On restriction of the follow-up time to 5 years the estimates were similar but the trend tests not significant

Overall, the relative risk of subsequent invasive breast cancer was almost twice as high for women under 40 years old at the first *in situ* breast cancer diagnosis compared with women over 40 years, with SIRs of 8.5 (95 % CI 6.1–11.7) and 4.4 (95 % CI 4.1–4.8), respectively (*P* value <0.001). Among women below 40 years, and who had a positive family history, the risk of subsequent invasive cancer was more than 14 times higher than in the general population, with a SIR of 14.3 (95 % CI 7.4–25.0).

Given that the background rates of breast cancer are highly age-dependent, we estimated the EAR in relation to age at diagnosis. While the relative risk of a subsequent invasive breast event decreased with increasing age, in both women with and without a family history of breast cancer, the overall EAR was significantly increased but was similar in women below 40 years of age at diagnosis (93.2 per 10,000 person-years; 95 % CI 63.4–129.8) as compared to women over 40 (88.5 per 10,000 person-years; 95 % CI 80.4–97.0) (Additional file 1: Table S1). In contrast, women with a family history of breast cancer had the highest EAR, with women under 40 years of age carrying the greatest EAR (154.1 per 10,000 person-years; 95 % CI 77.1–266.3), compared to women older than 40 years at diagnosis (105.7 per 10,000 person-years; 95 % CI 78.9–136.8). This suggests that both relative and absolute risks are higher with younger age of onset of *in situ* disease in women with a positive family history.

Finally, regardless of family history the risk of subsequent invasive cancer in the first 5 years after first *in situ* breast cancer was increased more than fivefold compared to the general population (SIR 5.2; 95 % CI 4.7–5.7). In women with no family history there was a significant decline in both the relative and absolute risk over time, but this was not observed in women with a family history (Table 3 and Additional file 1: Table S1).

### Mortality

The overall risk of death in women with *in situ* cancer was significantly increased by 30 % compared to the general population but was highly dependent on the occurrence of a second invasive cancer event (Table 4). Women who did not have a second invasive event following *in situ* breast cancer, had a similar risk of death to women in the background population (SMR 1.0 (95 % CI 1.0–1.1)). In contrast, women who were diagnosed with an invasive breast cancer event after *in situ* breast cancer were twice as likely to die as compared to women in the general population (SMR 2.1 (95 % CI 1.7–2.4)), with no significant differences between women with and without a family history of breast cancer.

**Table 4:** Standardized mortality ratio (SMR) with second breast events (contralateral *in situ* or ipsilateral or contralateral invasive breast cancer) after diagnosis of first *in situ* breast cancer and its 95 % CI, by type of second breast event and family history

	All		No family history		Family history		<50		>50	
	Deaths (*n*)	SMR	Deaths (*n*)	SMR	Deaths (*n*)	SMR	Deaths (*n*)	SMR	Deaths (*n*)	SMR
	(95 % CI)		(95 % CI)		(95 % CI)		(95 % CI)		(95 % CI)	
Overall	1343	1.28	1258	1.2	85	1.44	122	2.19	1221	1.24
	(1.2–1.2)		(1.2–1.4)		(1.2–1.8)		(1.8–2.6)		(1.2–1.3)	
No event + 2^nd^ contralateral *in situ*	927	1.01	875	1.0	52	1.02	58	1.17	869	1.00
	(1.0–1.1)		(0.9–1.1)		(0.8–1.3)		(0.8–1.5)		(0.9–1.1)	
Second invasive^a^	132	2.06	122	2.03	10	2.54	29	8.03	103	1.7
	(1.7–2.4)		(1.7–2.4)		(1.2–4.7)		(5.4–11.5)		(1.4–2.1)	
Second ipsilateral invasive	63	2.16	58	2.12	5^b^	2.75	17	12.89	46	1.65
	(1.7–2.8)		(1.6–2.7)		(0.9–6.4)		(7.5–20.6)		(1.2–2.2)	
Second contralateral invasive	55	1.99	49	1.92	6^b^	2.82	10	7.85	45	1.71
	(1.5–2.6)		(1.4–2.5)		(1.0–6.2)		(3.8–14.4)		(1.2–2.3)	

^a^Includes ipsilateral, contralateral and missing side. ^b^One subject had both ipsilateral and contralateral invasive breast cancer, which is why the total is 6 + 5 = 11 > 10

The overall risk of death following *in situ* breast cancer was increased in women with a family history (SMR1.4 (95 % CI 1.2–1.8)) and in women without family history (SMR 1.3 (95 % CI 1.2–1.4)). Given that deaths were rare at younger ages, we compared mortality among women above and below age 50 years. Women below age 50 years at the first *in situ* breast cancer diagnosis and who were diagnosed with a second invasive cancer, had significantly higher mortality as compared to women over 50 years at diagnosis (SMR 8.0; 95 % CI 5.4–11.5 vs. SMR 1.7; 95 % CI 1.4–2.0). The laterality of the second invasive event did not influence the risk of death significantly.

## Discussion

In this large population-based cohort, with data from nationwide, high-quality registers, we demonstrate that women diagnosed with *in situ* breast cancer have a considerably increased risk of invasive breast cancer and contralateral *in situ* breast cancer, compared to women in the general population, with young women facing the highest risk. Having a positive family history increases the risk exclusively for a contralateral invasive breast cancer by 50 % compared to not having a family history of breast cancer. The increased risk of invasive cancer persists over time, and at fifteen years after diagnosis the risk is still three times higher than in women in the general population. Meanwhile, the mortality for women with *in situ* breast cancer is the same as the general population, as long as invasive cancer does not occur.

In women with a positive family history, the risk of contralateral invasive breast cancer was more than four times as high as for women in the general population and almost 50 % higher compared to women with no family history of breast cancer. The observed increased risk is approximately twice as high as the risk of breast cancer that is faced by women without previous breast cancer, who have a positive family history of breast cancer. There are methodological issues that may account for these differences, because our estimates assume only one breast is at risk, with a corresponding lower expected rate.

Two meta-analyses of familial risks of breast cancer report the relative risk associated with having a first-degree relative with breast cancer as 2.1 and 1.8, respectively [28, 29]. The observed diluted additional risk in women with a family history, i.e., only 50 % increased risk of a contralateral invasive cancer, and no increased risk for ipsilateral invasive cancer or contralateral *in situ* cancer, as compared to women with no family history, is intriguing. We speculate that women with a positive family history were likely more prone to choose mastectomy than those without family history, which would reduce the risk of an ipsilateral cancer in these women. The reduced risk may also be a reflection of heterogeneity of the *in situ* breast cancer phenotype. Additional stratification into one, two or even three affected first-degree members to better quantify the hereditary component may have allowed a deeper understanding of these results.

Regardless of family history, women under 40 years of age at diagnosis had a significantly higher risk of subsequent invasive breast cancer compared to women above 40 years. These young women would experience an absolute excess risk ranging from about 8 events per 1000 person-years to as many as 15 events per 1,000 person-years depending on family history; this absolute excess risk decreases with increasing age only in women with a positive family history. Given that a younger woman with both high risk of a subsequent event and a longer life expectancy, which translates to higher cumulative risk, mastectomy may be considered to a greater extent in this patient population.

The increased relative risk of subsequent invasive breast cancer by almost 60 % from the period 1980–1984 to the period 2000–2004, exclusively in women with no family history, may be related to a combination of screening and treatment patterns. A nationwide mammography screening program was introduced during the study period, which had complete national coverage by 1997 [30]. Thus, the means of detection of *in situ* breast cancer changed during the study period, from symptom-detected to screening-detected, with better prognosis for the latter [31]. However, in this study the risk of subsequent invasive breast cancer increased during the study period and this might reflect that with increasing mammography screening and subsequently a larger number of smaller lesions detected, the use of breast-conserving surgery became the norm from 1990 onwards [32]. In comparison to mastectomy, breast-conserving surgery poses an increased risk of both local recurrence and new ipsilateral primary cancer. In contrast, women with a positive family history had no increased risk during the study period and we speculate that these women, who had relatives with breast cancer, were more prone to choosing mastectomy.

Treatment for *in situ* breast cancer during the study period was performed according to regional and national guidelines [32]. Surgery involved either mastectomy or breast-conserving surgery, and since the 1990s breast-conserving surgery for *in situ* breast cancer has been recommended whenever feasible. Several randomized trials, including the Swedish National DCIS study, have unanimously shown a decreased rate of ipsilateral *in situ* or invasive breast cancer recurrence through the addition of adjuvant radiotherapy after breast-conserving surgery [19, 33–37], and today, national guidelines include radiotherapy up to a total of 50 Gray after breast-conserving therapy in patients with ductal carcinoma *in situ* [32]. However, during most of the study period adjuvant radiotherapy was not recommended for the majority of patients who were treated with breast-conserving surgery, which might reflect the increased relative risk of a second invasive event in the latter parts of the study period when breast-conserving surgery become more frequent.

During follow up, women with no family history of breast cancer had a gradually decreasing risk of subsequent invasive breast cancer with time since diagnosis. However, 15 years after the first *in situ* breast cancer, the risk for an invasive breast cancer was still almost three times higher than for women in the general population. This indicates that women diagnosed with *in situ* breast cancer have a lifelong increased risk, which needs to be taken into account when planning their follow up.

Overall, there was no increased risk of death for women with *in situ* breast cancer as long as there was no second invasive event, but in women with a second invasive breast cancer the risk of death was doubled. There were no significant differences in mortality between women with and without family history of breast cancer. Young age of onset was an important predictor of death for women with *in situ* disease due to an increased risk for second invasive cancers and thus a substantially higher mortality, which should be taken into account when planning their treatment and follow-up.

In women with elevated risk of breast cancer, studies have shown that adjuvant endocrine therapy with a selective estrogen receptor modulator or an aromatase inhibitor reduces the risk by 40–50 % [38–40], and in women with lobular and ductal cancer *in situ* some studies suggest that the benefits are even greater [12, 38, 41, 42]. Today, national Swedish guidelines do not support the use of adjuvant endocrine therapy after standard therapy for ductal cancer *in situ*, and for lobular cancer *in situ* surgical or adjuvant treatment is still not recommended. One must weigh the benefits of endocrine therapy in reducing second breast cancer events against an increased risk of side effects. In a systematic review and meta-analysis the number needed to treat in order for Tamoxifen to have a protective effect against all breast events was 15 and it did not reduce the risk of all-cause mortality [43].

Strengths of the current study include the population-based design, the large sample size, complete follow up and unbiased ascertainment of family history, cancers and death. To the best of our knowledge, this is the largest study to assess the impact of a positive family history of breast cancer on risk and mortality after *in situ* breast cancer.

This study has a number of limitations. We have no information on the mode of detection, tumor grade or adjuvant treatment, and have not distinguished between mastectomies and breast-conserving surgery, or ductal carcinoma *in situ* and lobular carcinoma *in situ*. With this stated, a previous Swedish case–control study has shown that the risk of a subsequent invasive breast cancer was equal after lobular and ductal carcinoma *in situ* breast cancer [17]. One study has shown that for women with lobular carcinoma *in situ*, family history does not increase the risk of invasive breast cancer [12], thus among women with ductal carcinoma *in situ* and a positive family history, the risk estimates might be higher than shown in our study. During the study period, Sweden did not have a nationwide register on local recurrences and in the vast majority of regions, a second ipsilateral *in situ* breast cancer event was not reported. Therefore, new ipsilateral *in situ* cancer was not included in the study, as these events most probably would be underestimated.

## Conclusions

Among women with *in situ* breast cancer, a positive family history of breast cancer increases the risk of contralateral invasive breast cancer by almost 50 %. The risk of subsequent invasive breast cancer and of mortality is substantially higher in younger women, which should be taken into account when planning their treatment and follow up.

## Additional file

Additional file 1: Table S1. Excess additive risks (EAR) per 10,000 person-years after diagnosis of first *in situ* breast cancer, presented stratified by year at first diagnosis, age at first diagnosis, time since first diagnosis and family history. (DOC 45 kb)

## Abbreviations

CI: confidence interval
EAR: excess additive risks
SIR: standardized incident ratio
SMR: standardized mortality ratio
