# Supplementary material for: The impact of in situ breast cancer and family history on risk of subsequent breast cancer events and mortality - a population-based study from Sweden
Source: Breast Cancer Res. 2016 Oct 18;18:105. doi: 10.1186/s13058-016-0764-7 (PMC5069805; doi:10.1186/s13058-016-0764-7)
Supplement: Additional file 1: Table S1. — Excess additive risks (EAR) per 10,000 person-years after diagnosis of first in situ breast cancer, presented stratified by year at first diagnosis, age at first diagnosis, time since first diagnosis and family history. (DOC 45 kb) [file 13058_2016_764_MOESM1_ESM.doc]

|  |  |  | | | | | |
| --- | --- | --- | --- | --- | --- | --- | --- |
|  |  |  | **All** | **No Family History** | | **Family History** | |
|  |  | **No. of cases** | **EAR**  **(95% CI)** | **No. of cases** | **EAR**  **(95% CI)** | **No. of cases** | **EAR**  **(95% CI)** |
| **Calendar**  **year*** | 1980-1984 | 81 | 52.75  (37.45,70.43) | 72 | 48.28 (33.04,66.05) | 9 | 110.89 (42.95,215.85) |
|  | 1985-1989 | 141 | 61.81  (48.36,76.82) | 123 | 57.73 (44.05,73.13) | 18 | 100.78  (51.95,167.20) |
|  | 1990-1994 | 292 | 107.88 (93.16,123.77) | 259 | 105.83  (90.45,122.51) | 33 | 116.53 (73.94,169.99) |
|  | 1995-1999 | 182 | 111.20  (92.24,132.08) | 160 | 109.53  (89.57,131.67) | 22 | 110.38  (61.01,175.62) |
|  | 2000-2004 | 72 | 109.35  (80.32,143.22) | 63 | 108.33 (77.66,144.49) | 9 | 114.67 (41.80,227.26) |
| **Age at**  **diagnosis** | < 40 | 39 | 93.17  (63.42,129.84) | 27 | 77.90  (47.93,116.43) | 12 | 154.10 (77.14,266.30) |
|  | 40-49 | 173 | 85.36  (70.19,102.11) | 147 | 84.82  (68.47,103.03) | 26 | 81.46  (46.61,126.48) |
|  | 50-59 | 221 | 83.61  (69.64,98.88) | 189 | 78.20  (63.88, 3.94) | 32 | 117.94 (73.05,174.48) |
|  | 60-69 | 220 | 98.46  (82.54,115.85) | 207 | 96.87  (80.69,114.59) | 13 | 121.11 (53.53,218.20) |
|  | ≥70 | 115 | 85.99  (66.78,107.69) | 107 | 82.72  (63.43,104.60) | 8 | 152.17  (55.54,305.46) |
|  | <40 | 39 | 93.17  (63.42,129.84) | 27 | 77.90  (47.93,116.43) | 12 | 154.10  (77.14,266.30) |
|  | >40 | 729 | 88.50  (80.41,96.99) | 650 | 85.87  (77.50,94.67) | 79 | 105.72 (78.88,136.82) |
|  | 0-4 | 401 | 98.39  (86.87,110.69) | 359 | 97.42  (85.34,110.38) | 42 | 98.33  (65.49,138.51) |
| **Time**  **since**  **diagnosis** | 5-9 | 230 | 88.49  (74.36,103.89) | 197 | 82.22  (67.83, 98.01) | 33 | 137.92  (88.46,200.00) |
|  | 10-14 | 96 | 66.28  (48.77,86.29) | 85 | 63.44  (45.47,84.15) | 11 | 89.41 (33.33,172.54) |
|  | 15+ | 41 | 69.06 (42.11,102.12) | 36 | 62.90 (36.15,96.16) | 5 | 143.72 (32.81,342.47) |
|  |  |  |  |  |  |  |  |

**Additional file 1: Table S1. Excess additive risks (EAR) per 10,000 person-years after diagnosis of first *in situ* breast cancer, presented stratified by year at first diagnosis, age at first diagnosis, time since first diagnosis and family history**
